# Nano-Based Drug Delivery of Polyphenolic Compounds for Cancer Treatment: Progress, Opportunities, and Challenges

**DOI:** 10.3390/ph16010101

**Published:** 2023-01-10

**Authors:** Wenhui Jia, Li Zhou, Lei Li, Ping Zhou, Zhisen Shen

**Affiliations:** 1 State Key Laboratory of Biotherapy and Cancer Center, West China Hospital, West China School of Basic Medical Sciences & Forensic Medicine, Collaborative Innovation Center for Biotherapy, Sichuan University, Chengdu 610041, China; 2 Key Laboratory of Molecular Biology for Infectious Diseases (Ministry of Education), Institute for Viral Hepatitis, Department of Infectious Diseases, The Second Affiliated Hospital, Chongqing Medical University, Chongqing 400016, China; 3 School of Basic Medical Sciences, Chengdu University of Traditional Chinese Medicine, Chengdu 611137, China; 4 Department of Radiotherapy, The First Affiliated Hospital of Hainan Medical University, Haikou 571199, China; 5 Department of Otorhinolaryngology and Head and Neck Surgery, The Affiliated Lihuili Hospital, Ningbo University, Ningbo 315211, China

**Keywords:** polyphenolic compounds, nanosystem, drug delivery, cancer treatment

## Abstract

Polyphenols and their derivates, a kind of natural product distributed in herb plants, vegetables, and fruits, are the most abundant antioxidants in the human diet and have been found to display cancer-preventative effects in several epidemiological studies. The scientific community has also validated the anti-cancer bioactivities and low toxicities of polyphenolic compounds, including flavones, tannins, phenolic acids, and anthocyanins, through in vitro and in vivo studies. However, the low stability, weak targeting ability, poor solubility, and low bioavailability of pure polyphenolic agents have significantly impaired their treatment efficacy. Nowadays, nano-based technology has been applied to surmount these restrictions and maximize the treatment efficacy of polyphenols. In this review, we summarize the advantages and related mechanisms of polyphenols in cancer treatment. Moreover, aiming at the poor solubility and low bioavailability of pure polyphenols in vivo, the advantages of nano-based delivery systems and recent research developments are highlighted. Herein, particular emphasis is mainly placed on the most widely used nanomaterials in the delivery of natural products, including liposomes, micelles, and nanogels. Finally, we present an overview and the challenges of future implementations of nano-based delivery systems of polyphenolic compounds in the cancer therapeutic field.

## 1. Introduction

**Figure 1:**
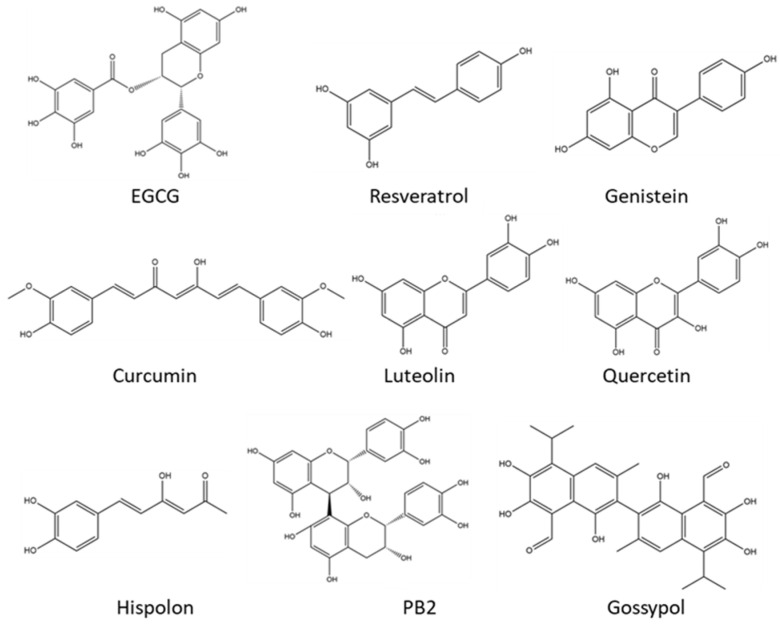
Chemical structures of some common polyphenolic compounds derived from natural products with antitumor activity.

Polyphenolic compounds, a class of plant-derived natural products with at least one membered aromatic ring and some hydroxyl groups [1,2,3], are primarily derived from secondary metabolites of plants and are widely distributed in daily dietary foods [4,5]. Polyphenolic compounds have extensive subtypes including flavones, tannins, phenolic acids, anthocyanins, etc. [6,7,8]. In ancient China, many herbal medicines, such as *S. glabra* (commonly called *Zhong Jie Feng* in Chinese) and *Xanthoceras sorbifolium* bunge, were used by numerous quack doctors to deal with abscesses, rheumatism, and other diseases, and polyphenols were later proved to be active ingredients in them [9,10]. Recently, pharmacological studies have demonstrated that polyphenolic compounds exhibit strong antioxidant effects and display obvious antitumor potential [11,12,13] (Figure 1). In the combat against tumors, much attention has been paid to the development of polyphenolic compounds sourced from natural products because of their low toxicities and abundance of resources [14,15]. However, polyphenolic compounds derived from natural products also have some drawbacks, such as low stability, weak targeting ability, and poor solubility and bioavailability, which limit their applications in the clinical treatment of cancers [16,17,18].

The recent development of nanotechnology has gained tremendous attention as an available avenue for overcoming these deficiencies of polyphenolic compounds in natural products. Nano-based drug delivery systems can improve the circulating time of drugs in plasma and promote their distribution [19]. Moreover, nanotechnology enables a targeted delivery that can precisely deliver drugs to tumor sites [20,21]. Tumor cells highly express many receptors/transporters specifically, and thus nanoparticles (NPs) engineered with targeting ligands can enter these cells selectively [22]. In addition, the tumor microenvironment (TME) has some specific characteristics, such as being weakly acidic, hypoxia, and so on [23,24]. These properties can be applied to the design of TME-responsive NPs that promote the precise uptake and release of drugs in tumor cells [25,26]. Nowadays, biomimetic nanocarriers, such as endogenous extracellular vesicles, have been vigorously developed to overcome the possible immunogenicity of chemically synthesized materials [27,28]. In addition, nano-based drug delivery systems provide the possibility for the co-delivery of natural products and chemotherapeutic drugs, which can enhance the antitumor effects [29,30].

In this review, we first provide a comprehensive overview of the anti-cancer properties of polyphenolic compounds sourced from natural products and the related molecular mechanisms. Afterward, we emphasize the advantages of nano-based delivery systems and review recent progress in the nanocarrier-mediated delivery of polyphenolic compounds in cancer treatment. Finally, we highlight the significance of nanotechnology in the delivery of polyphenolic compounds and discuss the latest insights in this field. Using evidence from the literature, numerous available inspirations can be provided for designing sophisticated and innovative nano-based delivery systems of polyphenolic compounds in the future.

## 2. Overview of Anti-Cancer Properties and Involved Mechanisms of Polyphenolic Compounds

Recently, tremendous basic and clinical studies have demonstrated that polyphenolic compounds extracted from natural products exhibit considerable antitumor activities. In this section, we look at recent progress in the study of how polyphenolic compounds affect cell growth, cell cycle, apoptosis, cell invasion, and metastasis (Table 1). This gives us a foundation for the rest of the paper.

### 2.1. Effects on Inhibiting Cell Proliferation and Cell Cycle

**Figure 2:**
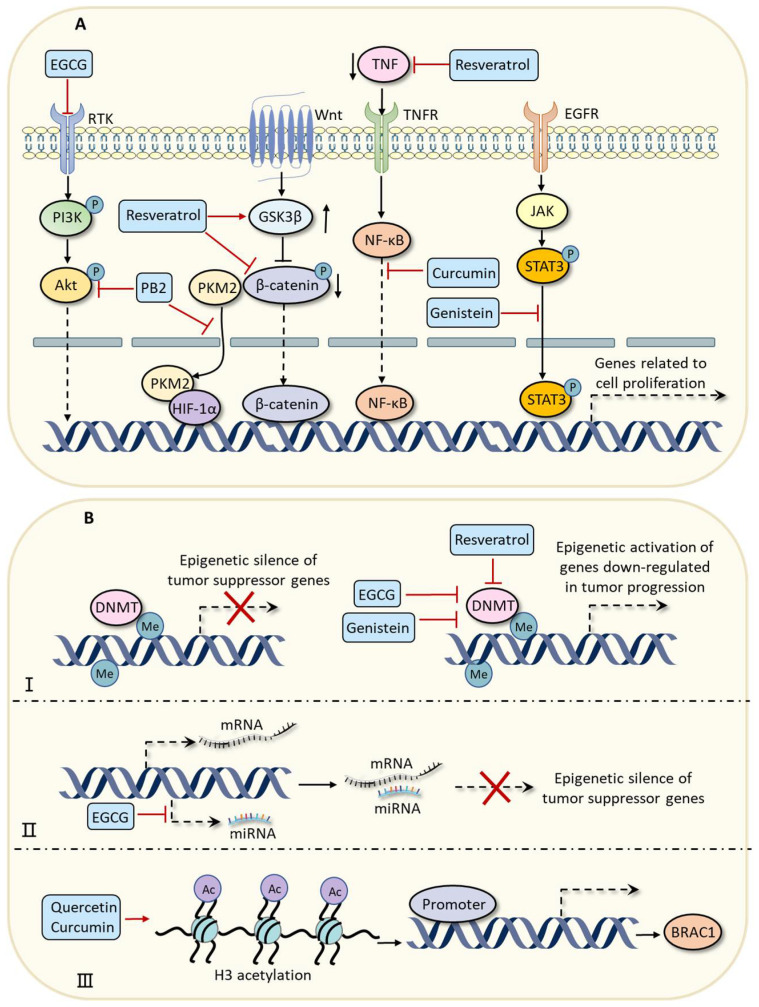
(**A**) Polyphenolic compounds can inhibit tumor growth by modulating various signaling pathways associated with cell proliferation and cell cycle. Polyphenolic compounds EGCG and PB2 can prevent the proliferation of cancer cells by modulating Akt/PI3K signaling pathway. PB2 can also inhibit the nuclear translocation of PKM2 and disrupt its interaction with HIF-1α to suppress the proliferation of cancer cells. Resveratrol can inhibit the proliferation of cancer cells by modulating Wnt/β-catenin and NF-κB signaling pathways. Curcumin can block the nuclear translocation of NF-κB to inhibit cancer development. Genistein can induce cell cycle arrest by inhibiting the JAK/STAT3 signaling pathway. (**B**) Polyphenolic compounds can inhibit tumor growth through epigenetic regulation. (I) Treatment with polyphenolic compounds EGCG, genistein, or resveratrol can suppress the activity of DNMT and decrease DNA methylation levels, thereby recovering the transcriptional activity of tumor suppressor genes. (II) EGCG can inhibit the expression of miRNAs that target tumor suppressor genes. (III) Quercetin and curcumin can enhance the expression of BRAC1 by increasing the acetylation of histone H3K9 in the promoter of BRCA1 gene. Abbreviations: EGCG, epigallocatechin-3-gallate; Akt, protein kinase B; PI3K, phosphatidylinositide 3-kinase; PB2, proanthocyanidin-B2; PKM2, pyruvate kinase M2; HIF-1α, hypoxia-inducible factor 1α; TNF, tumor necrosis factor; NF-κB, nuclear factor kappa-B; GSK3β, glycogen synthase kinase-3β; STAT3, signal transducer and activator of transcription-3; JAK, janus-activated kinase; DNMT, DNA methyltransferase; BRAC1, breast cancer 1.

Epigallocatechin-3-gallate (EGCG) is a polyphenolic compound sourced from green tea that possesses many biological activities, such as antitumor and anti-inflammatory characteristics [56]. Studies have shown that EGCG can prevent the proliferation of prostate cancer cells and breast cancer cells by modulating the protein kinase B (Akt)/phosphatidylinositide 3-kinases (PI3K) signaling pathway, which participates in the transcriptional regulation of many genes related to cancer cell proliferation [31,56]. Mechanistically, EGCG can interact with receptor tyrosine kinases (RTKs), which play a key role in the activation of the Akt/PI3K signaling pathway [57]. In addition, polyphenolic proanthocyanidin-B2 (PB2), usually found in grape seeds and peanut skin, was shown to have the ability to suppress the proliferation of liver cancer cells and hepatocellular carcinogenesis by directly binding to Akt and inhibiting the activation of the Akt/PI3K signaling pathway [35]. Additionally, PB2 has been demonstrated to inhibit the nuclear translocation of pyruvate kinase M2 (PKM2) and disrupt its interaction with hypoxia-inducible factor 1α (HIF-1α), thereby suppressing proliferation and triggering apoptosis in hepatocellular carcinoma via transcriptional regulation [36]. Resveratrol is a non-flavonoid polyphenolic compound present in grapes, berries, and soybeans [58]. It was demonstrated to have the ability to inhibit the proliferation of breast-cancer-like stem cells by modulating the Wnt/β-catenin signaling pathway [37]. Moreover, resveratrol can decrease the expression of tumor necrosis factor (TNF)-β to suppress the proliferation of tumor cells by preventing the nuclear translocation of nuclear factor kappa-B (NF-κB) [38]. In addition, curcumin is a natural polyphenolic compound extracted from the rhizome of Curcuma longa (turmeric), and researchers have demonstrated that it has many positive biological activities in relation to oxidative stress and inflammation [59,60]. Curcumin was demonstrated to inhibit the NF-κB signaling pathway in cervical cancer cells [43]. In addition to these compounds, genistein, a predominant isoflavonoid commonly originating from legumes and dentate plants, was suggested to have the ability to block the Janus-activated kinase 1/2 (JAK1/2)-signal transduction and the signal transducer and activator of transcription 3 (STAT3) signaling pathways in esophageal carcinoma cells by suppressing the expression of epidermal growth factor receptor (EGFR), which finally leads to the inhibition of cell proliferation and cell cycle arrest [46]. Hispolon is a natural polyphenolic compound usually derived from the traditional medicinal mushroom *phellinus linteus* [61]. Some researchers have demonstrated that hispolon can induce cell cycle arrest in various cancers [62]. Specifically, hispolon can induce cell cycle G2/M arrest in glioblastoma cells by suppressing the expressions of cyclin B1, cell division cycle 2 (CDC2), and M-phase inducer phosphatase 3 (CDC25C), which are three major regulatory proteins of cell cycle [63]. In summary, these studies demonstrate that polyphenolic compounds can inhibit the proliferation of cancer cells and promote cell cycle arrest by modulating associated pathways, such as the Akt/PI3K, Wnt/β-catenin, NF-κB, and JAK1/2-STAT3 signaling pathways (Figure 2).

Some natural polyphenolic compounds may also inhibit tumor growth through epigenetic regulation. For example, EGCG can regulate the expression of genes associated with cell proliferation and invasion by modifying DNA methylation and chromatin remodeling in breast cancer cells [49]. Specifically, EGCG can suppress the activities of DNA methyltransferases (DNMTs) and histone deacetylases (HDACs), thereby recovering the expression of tumor suppressor genes [32,49]. In myeloma cells, treatment with EGCG was reported to downregulate the expressions of miR-25, miR-92, miR-141, and miR-200a [33]. These miRNAs can target the tumor suppressor gene p53 and decrease its expression [33]. This study showed that EGCG could inhibit the proliferation of cancer cells by recovering the activity of tumor suppressor gene p53. Genistein has also been shown to decrease the CpG methylation in promoters of tumor suppressor gene breast cancer 1 (BRAC1), which is usually silenced in triple-negative breast cancer [64], leading to decreased cell proliferation in breast cancer cells [47]. It was also revealed by others that treatment with quercetin and curcumin could enhance the expression of BRAC1 by increasing the acetylation of histone H3K9 in the promoter of the BRCA1 gene to inhibit the proliferation of breast cancer cells [49]. Moreover, genistein treatment can inhibit the promoter methylation of various tumor suppressor genes induced by bisphenol A (a carcinogen in various plastics such as food containers) in breast cancer cells [65]. Sarcoplasmic/endoplasmic reticulum calcium ATPase 3 (ATP2A3) is a significant component in the Ca^2+^ signaling network, which participates in regulating various cellular processes such as differentiation, proliferation, and cell death [66]. Recent studies demonstrated that ATP2A3 was downregulated in various cancers [67]. Resveratrol can enhance ATP2A3 expression through epigenetic modification on the promoter and suppressing the activity of DNMT [39].

### 2.2. Effects on Inducing Autophagic or Apoptotic Cell Death

**Figure 3:**
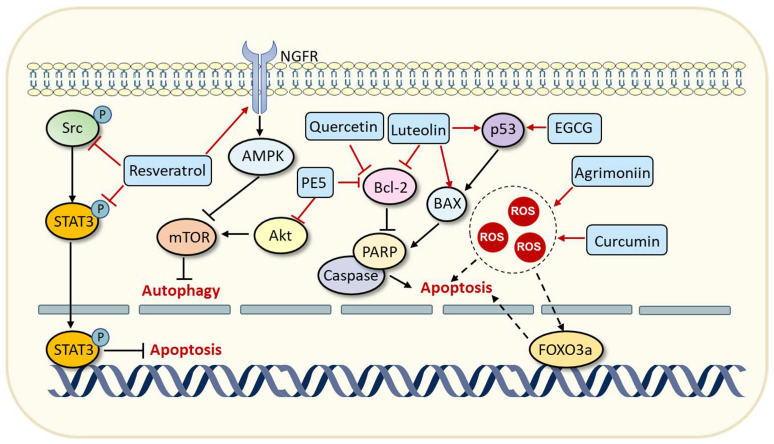
Polyphenolic compounds can trigger apoptosis and autophagy of cancer cells. Resveratrol can induce autophagy of cancer cells by modulating the AMPK/mTOR signaling pathway while inducing apoptosis by inhibiting the Src-STAT3 signaling pathway. The polyphenolic compound PE5 can trigger autophagy and apoptosis by intervening Akt/mTOR and Bcl-2 signaling pathways. Luteolin could induce apoptosis by increasing the expressions of p53 and BAX while decreasing the level of Bcl-2. Similarly, the polyphenolic compound EGCG was demonstrated to induce apoptosis by increasing the stability and transcriptional activity of tumor suppressor p53. Quercetin can trigger apoptosis in cancer cells by decreasing the expression of Bcl-2. Treatment with polyphenolic compounds agrimoniin and curcumin can lead to intracellular ROS accumulation, thereby triggering apoptosis of cancer cells. Abbreviations: EGCG, epigallocatechin-3-gallate; Akt, protein kinase B; STAT3, signal transducer and activator of transcription-3; AMPK, adenosine 5′-monophosphate (AMP)-activated protein kinase; mTOR, mammalian target of rapamycin; ROS, reactive oxygen species; FOXO3a, forkhead box protein O3a; Bcl-2, B-cell lymphoma-2; PE5, 2-(4″-hydroxybenzyl)-5-2″-dihydroxy-3-methoxystilbene; BAX, Bcl-2-associated X protein; PARP, poly ADP-ribose polymerase.

Adenosine 5′-monophosphate (AMP)-activated protein kinase (AMPK)/mammalian target of rapamycin (mTOR) and Akt/mTOR are two crucial signaling axes in the regulation of apoptosis and autophagy, so they are often used as targets for antitumor therapy. A natural polyphenolic compound 2-(4″-hydroxybenzyl)-5-2″-dihydroxy-3-methoxystilbene (PE5) isolated from the roots of the *Phragmipedium* species was verified to trigger autophagy and apoptosis in lung cancer cells by intervening Akt/mTOR and B-cell lymphoma-2 (Bcl-2) signaling pathways [51]. In addition, a thermostable flavonoid, luteolin, could induce apoptosis in breast cancer cells by increasing the expressions of p53 and Bcl-2-associated X protein (BAX) while decreasing the level of Bcl-2 [52]. Similarly, the polyphenolic compound EGCG was demonstrated to induce apoptosis by increasing the stability and transcriptional activity of tumor suppressor p53 in prostate cancer cells [68]. Quercetin can trigger apoptosis in cancer cells by decreasing the expression of Bcl-2 via a mitochondria-mediated pathway [50]. Moreover, some researchers discovered that quercetin treatment can also trigger protective autophagy by modulating Akt/mTOR signaling and activating HIF-1α signaling, which counteracted quercetin-mediated apoptotic cell death and impaired its therapeutic efficacy [50]. Eukaryotic Initiation Factors 2α (eIF2α) is a crucial regulatory subunit in the translation process of protein synthesis in eukaryotic cells [69]. Many studies have shown that the abnormal fluctuation of the phosphorylation level of eIF2α is associated with the proliferation and invasiveness of tumor cells [70,71]. A combinational treatment of resveratrol and cisplatin can increase the level of phosphorylated eIF2α, which leads to increased intracellular Ca^2+^ levels, thereby triggering endoplasmic reticulum stress and apoptosis of gastric cancer cells [72]. Alongside these treatments, the accumulation of intracellular reactive oxygen species (ROS) has also been shown to be associated with the induction of apoptosis [73]. Agrimoniin is a type of polyphenolic compound derived from *Agrimonia pilosa ledeb*, a perennial herb that has been widely used in traditional Chinese medicine [74]. A study has shown that agrimoniin can significantly increase intracellular ROS levels and lead to the dysfunction of mitochondria, which finally triggers apoptosis of pancreatic cancer cells [53]. Forkhead box O3 (FOXO3a) belongs to the family of forkhead transcription factors that plays a significant role in general cellular processes, such as proliferation, apoptosis, differentiation, and DNA damage repair [75]. FOXO3a is abnormally downregulated in various cancers for it can induce apoptosis or exert other tumor-suppressive effects [75,76]. Curcumin and its analogs have been shown to increase the expression of FOXO3a by enhancing ROS generation in lung cancer cells [77]. Additionally, polyphenolic compound resveratrol was demonstrated to induce the apoptosis of cancer cells by suppressing the phosphorylation of the Src-STAT3 signaling pathway [40,78]. Resveratrol can also induce the autophagy of cancer cells by modulating the AMPK/mTOR signaling pathway [41]. Taken together, these studies indicate that polyphenolic compounds derived from natural products can induce autophagic or apoptotic cell death by regulating different pathways (Figure 3).

### 2.3. Suppressing Cell Invasion and Metastasis

**Figure 4:**
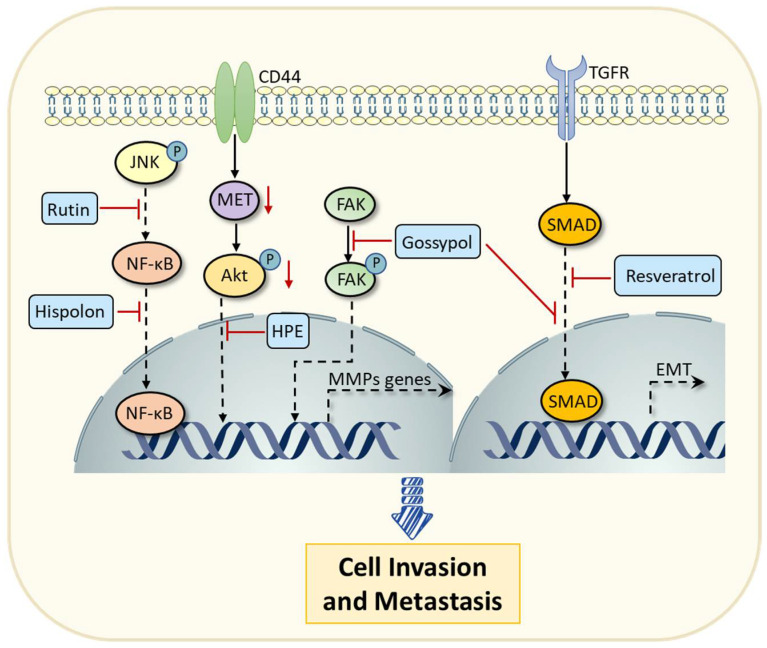
Polyphenolic compounds (e.g., rutin, hispolon, HPE, and gossypol) can inhibit metastasis and invasion of cancer cells by downregulating the expression of MMPs via various signaling pathways, including NF-κB, CD44/c-MET, and FAK signaling pathways. Gossypol and resveratrol can also reverse the EMT mediated by the TGF-β/Smad signaling pathway to suppress the metastasis and invasion of cancer cells. Abbreviations: JNK, c-Jun N-terminal kinase; NF-κB, nuclear factor kappa-B; HPE, *Hibiscus sabdariffa extract*; CD44, cluster of differentiation-44; c-MET, cellular-mesenchymal epithelial transition; FAK, focal adhesion kinase; EMT, epithelial–mesenchymal transition; MMPs, matrix metalloproteinases.

Rutin is a glycosylated form of quercetin and widely exists in many fruits and vegetables, such as citrus, onions, and mulberries [79]. Recent research has shown that rutin has the potential to be an effective metastatic inhibitor in the progression of cancer. Treatment with rutin can decrease the levels of matrix metalloproteinases (MMPs) by inhibiting the activation of the mitogen-activated protein kinase (MAPK)/NF-κB signaling pathway [80]. MMPs are a kind of endopeptidase enzyme that play a role in the degradation of the extracellular matrix (ECM), which is a crucial process during tumor invasion [81]. Consistently, the polyphenolic compound hispolon can suppress the expression of MMP-9 by inhibiting the NF-κB signaling pathway, thereby decreasing the invasive capabilities of breast cancer cells [48]. Polyphenolic compounds extracted from *Hibiscus sabdariffa* (HPE) were demonstrated to suppress colon carcinoma metastasis via inhibiting the cluster of differentiation-44 (CD44)/cellular–mesenchymal epithelial transition (c-MET) signaling pathways to decrease the expression of MMPs [54,82]. Moreover, a polyphenolic compound derived from cottonseed termed gossypol [83] has also been found to exhibit a strong suppressive effect on the metastasis of human cervical cancer cells [55]. Specifically, gossypol can reduce the expression of MMPs by inhibiting the focal adhesion kinase (FAK) signaling pathway, and on the other hand, it can reverse the epithelial–mesenchymal transition (EMT) mediated by transforming growth factor (TGF)-β [55]. Similarly, the natural polyphenolic compound resveratrol has also been shown to prevent EMT by inhibiting the TGF-β/Smad signaling pathway and downregulating the expression of transcription factor Snail [42]. In conclusion, these studies indicate that polyphenolic compounds derived from natural products can regulate cancer progression via multiple aspects, including cell proliferation and cell cycle, apoptosis and autophagy, and invasion and metastasis (Figure 4).

### 2.4. Other Involved Mechanisms

Accumulating evidence has indicated that polyphenolic compounds display some modulatory effects on gut microbiota, which can influence the development of colorectal cancer. Supplementation of polyphenolic compounds, such as isoliquiritigenin extracted from traditional Chinese medicine, anthocyanin derived from black raspberry, and EGCG, can influence the gut microbial composition of mice with colorectal cancer, making them more similar to those of healthy mice [34]. Polyphenolic compounds, in addition to tumor cells, can regulate the behaviors of cells in the TME, inhibiting tumor cell growth and proliferation indirectly. Cancer-associated fibroblasts (CAFs) are one of the main components of the TME and play a key role in promoting the progression and invasion of tumor cells by constructing a pro-inflammatory and immunosuppressive TME [84]. Curcumin, a polyphenolic compound, can inhibit prostate cancer cell growth and metastasis by inducing apoptosis in CAFs via activation of the ROS-mediated endoplasmic reticulum stress signaling pathway [45]. Castalagin is a polyphenolic compound derived from the berry Camu-camu (*Myrciaria dubia*) [85]. Some researchers discovered that oral administration of castalagin can improve the level of functional CD8+ T cells in the TME through the recruitment of gut bacteria related to efficient immune response (*Ruminococcaceae* and *Alistipes*), which enhanced the efficacy of anti-programmed death-1 (PD-1) therapy in cancer treatment [12]. Some epidemiological studies also demonstrated that polyphenolic compounds derived from coffee, such as ferulic acid, 3,4-dihydroxyphenylpropionic acid, and caffeic acid, showed therapeutic effects on colorectal cancer [86]. Polyphenolic compounds in green tea extracts can exert a suppressive effect on many types of tumors, including lung, stomach, pancreatic, prostate, esophagus, and breast cancers [87].

## 3. Advantages of Nano-Based Delivery Systems for Polyphenolic Compounds in Cancer Therapy

Although natural polyphenolic compounds have excellent tumor-suppression effects, poor solubility has restricted their clinical applications. Moreover, direct injection of these natural polyphenolic compounds into the bloodstream may lead to severe adverse effects. In addition, their short circulation time and rapid metabolism also affect their therapeutic efficacy. Due to these problems, researchers started to explore new delivery strategies to maximize the treatment outcomes of various therapeutic polyphenolic compounds. Currently, it has been demonstrated that the emerging field of nanotechnology has the potential to improve the delivery efficiencies and cancer treatment outcomes of many chemotherapeutic drugs, antitumor vaccines, and nucleic acids [88,89,90,91]. Nano-based drug delivery systems can improve many natural drawbacks of polyphenolic compounds due to their structural properties. According to that, we summarize the advantages of nanotechnology in the delivery of natural polyphenolic compounds in cancer treatment.

### 3.1. Increasing the Aqueous Solubility via Nanomaterials

Wrapping water-insoluble natural polyphenolic compounds into hydrophilic nanomaterials can improve the delivery efficiency and cellular uptake of drugs [92]. Quercetin has shown excellent antitumor activities in many types of cancer, for example, it can induce apoptosis in leukemia and trigger cell cycle arrest in prostate cancer [93,94]. However, problems such as poor aqueous solubility make quercetin an unreliable choice for clinical cancer treatment [95]. The advent of nanotechnology has expanded the prospects for the clinical application of quercetin. Sun et al. reported a co-delivery nanosystem of ginsenoside Rg3 and quercetin that is hydrophilic and can be used for intravenous administration with good biosafety [96]. Moreover, the concentration of free drugs in the plasma decreased quickly while drugs in the nanosystem stayed significantly longer in the plasma [96]. Kaempferol, a polyphenolic compound with antitumor activity, also faces the challenge of poor solubility [97,98]. A recent study reported a nanosystem incorporated with kaempferol that had excellent antitumor efficacy via disrupting calcium homeostasis in cancer cells [99]. In this study, kaempferol was loaded into CaCO_3_ NPs and encapsulated with the membrane of a human pulmonary carcinoma (A549) cell to achieve targeted delivery [99]. The authors first verified that the proliferation suppressive ability of this nanoplatform was stronger than that of pure kaempferol in in vitro cell experiments [99]. They also detected the expression of apoptosis-related proteins and preliminarily explored the related mechanisms [99]. Importantly, this nanosystem overcomes the poor solubility and bioavailability of kaempferol and exhibits good in vivo administration in cancer treatment [99]. Genistein is a natural polyphenolic compound with strong antioxidant and anti-inflammatory bioactivities, which afford it a good antitumor performance [100]. However, its poor water solubility and rapid metabolism restrict its application, making the plasma or tissue concentrations of genistein in vivo much lower than its in vitro IC50 [101,102]. Gold NPs can increase the bioavailability of genistein and provide a possible delivery strategy to efficiently preserve the antitumor performance of genistein in vivo [102]. The in vivo antitumor performance of this nanosystem is even better than that observed in in vitro cell experiments because of the good targeting ability of this nanoplatform mediated by enhanced permeability and retention (EPR) effects [102]. Moreover, many researchers used water-soluble and harmless shell materials (e.g., modified starch, gum, and maltodextrin) to encapsulate anti-cancer polyphenolic compounds with poor solubility, which allowed better bioavailability in vivo [16]. Some researchers also hope to improve the water solubility and stability of resveratrol through the synthesis of resveratrol-modified mesoporous silica NPs [103]. These NPs can induce apoptosis in gastric cancer cells and inhibit tumor growth in vivo with no obvious adverse effects on normal tissues and organs, which greatly broadens the clinical application potential of resveratrol [103]. Additionally, the application of natural products via nanoplatforms can optimize the chemo-physical properties of some frequently-used chemotherapeutic agents and improve their therapeutic performance in vivo. A tannic acid–docetaxel self-assemblies nanoplatform was developed recently [104]. Tannic acid is a polyphenolic natural compound with anti-cancer abilities against various cancers, such as breast cancer and prostate cancer [105]. Incorporating tannic acid into the nanosystem can facilitate the solubilization of docetaxel [104]. In addition, this nanoplatform was demonstrated to be an efficient delivery strategy to deliver chemotherapeutic docetaxel to prostate cancer cells, which greatly enhanced the treatment outcome [104].

### 3.2. Enhancing the Targeting Ability of Polyphenolic Compounds

Cancer cells highly express a large number of unique receptors on their surface, such as transferrin receptor 1 (TfR1) and CD44 [106,107]. Nanocarriers engineered with specific ligands to highly-expressed receptors on the cell membranes of cancer cells can achieve targeted drug delivery [108]. A kind of dextran-modified quercetin-Cu(II)/hyaluronic acid nanomedicine was developed to broaden the application prospect of quercetin in the treatment of triple-negative breast cancer [109]. This nanoplatform was smartly decorated with hyaluronic acid, a specific ligand for CD44, to achieve targeted delivery in the treatment of cancer [109]. Similarly, hyaluronic acid cross-linked zein nanogels were constructed to deliver curcumin in the treatment of colon cancer [110]. The nanogel can achieve targeted delivery via CD44-mediated mechanisms and improve the biocompatibility of curcumin, thereby enhancing the therapeutic effects [110]. Hu et al. reported a kind of lipid–calcium NP loaded with quercetin phosphate, which can be transformed into quercetin and exert antitumor effects under physiological conditions [111]. This nanoplatform was further encapsulated by lipid bilayers engineered with a tumor-specific targeting ligand aminoethylanisamide to enhance the tumor tissue targeting ability of quercetin phosphate [111]. In recent years, there have also been several studies about the utilization of cellular membranes in nano-based drug delivery systems. NPs modified with cellular membranes are more biocompatible and have exhibited some desirable characteristics inherited from source cells [112]. Moreover, many studies have shown that NPs decorated with cancer cell membranes have better tumor tissue targeting abilities and longer circulating times in vivo [113]. Based on this, a nanoplatform consisting of a core of mesoporous silica nanoparticles (MSNs) loaded with quercetin and a shell of cancer cell membranes was designed to achieve targeted therapy [112].

Moreover, the physicochemical properties (such as acidity and oxygen level) of the TME are very different from those of normal tissues, which makes it possible to design TME-responsive nanosystems [114,115]. In addition, the suitable sizes of NPs can promote the accumulation of drugs in tumor sites and increase the take-up efficiency of cancer cells via EPR effects [116]. Therefore, the application of nanotechnology can enhance the targeting ability and tumor accumulation of natural polyphenolic compounds, thus improving their antitumor performances. Because of the high glucose requirement, many glucose transporters are overexpressed in cancer cells [117]. Accordingly, glycol-conjugated biomaterials can be used to achieve targeted delivery of drugs to tumor cells. Levan is a natural polysaccharide derived from *Zymomonas mobilis*, and it can be used as an active tumor-targeting carrier due to the interactions between its glycosidic structure and glucose transporters on the membranes of cancer cells [118]. A study reported that a nanosystem using levan as the nanocarrier and loaded with curcumin, a natural polyphenolic compound with antitumor bioactivity, can achieve targeted delivery in the treatment of breast cancer [118].

### 3.3. Taking Advantage of the Structural Properties of Polyphenolic Compounds

Polyphenolic compounds can be integrated into some nanosystems to exert antitumor effects based on their inherent structural characteristics. Nanotechnology can precisely engineer drugs or carriers to enable them to have excellent therapeutic effects without systemic toxicity in vivo. Wu et al. reported a smart-engineered strategy for EGCG to optimize its delivery efficacy and immunotherapeutic effect [119]. They synthesized fluorinated-coordinative-EGCG, which had a higher stability and transfection efficacy than free EGCG due to the lipophobic and hydrophobic properties of fluorination [119]. Moreover, zinc ions can be incorporated into this nanosystem to increase the affinity of nanocarriers with cargos [119]. Phenolic ligands in polyphenolic compounds can coordinate with metal ions to constitute metal–phenolic networks, which can encapsulate chemotherapeutic agents and then release these functional components at tumor sites due to the weakly acidic TME [120]. A natural polyphenolic compound derived from the cotton plant, gossypol, self-assembly polyethylene glycol-chlorin e6 (PEG-Ce6) polyphenol, and Fe^2+^ were designed to fabricate a metal–phenolic network and form a nanoplatform that showed chemotherapeutic effects and significantly improved the treatment outcome of programmed death-ligand 1 (PD-L1) checkpoint blockade immunotherapy [121]. Yan and his colleagues used Mn^2+^ and amphiphilic polyethylene glycol (PEG)-polyphenol to construct metal–phenolic networks [122]. The nanosystem also incorporated a radiosensitizer via coordination with Mn^2+^ [122]. This nanosystem can inhibit tumor growth effectively via sensitizing radiation and triggering stimulator of interferon genes (STING)-pathway-mediated immunostimulation [122]. The construction of platinum-based nanomedicines often takes advantage of the coordination properties of Pt and polyphenolic compounds. Tannic acid and the prodrug of oxaliplatin were demonstrated to form a well-defined nanosystem for oxaliplatin delivery and cancer therapy [123]. Specifically, this nanosystem can not only promote the apoptosis of tumor cells but also enhance antitumor immune responses by promoting the recruitment of cytotoxic T cells in the TME, thereby achieving good antitumor outcomes synergistically [123]. Recent studies demonstrated that quercetin could inhibit the interaction of PD-1/PD-L1 to relieve immunosuppression in the TME [124] and remodel the TME by reducing the α-SMA+ fibroblast populations at tumor sites [111]. Moreover, natural polyphenolic compound quercetin can also coordinate with metal ions [125]. Therefore, some researchers designed quercetin–ferrum NPs to enhance the photothermal therapeutic effects and prevent the reoccurrence of melanoma by reducing immunosuppression and reshaping the TME [125].

In addition to traditional nano-based drug delivery systems, some natural polyphenolic compounds can promote the cross-linking of polymer chains, which plays a critical role in the formation and maintenance of functional hydrogels [126]. Incorporating the natural polyphenolic compound tannic acid can enhance the cross-linking of polymer chains, thereby increasing the stability of nanogels [127]. Moreover, tannic acid can coordinate with various metal ions and assist the loading of metal-containing agents such as the chemotherapeutic drug cisplatin [127]. Importantly, this tannic-acid-incorporated nanogel can also achieve acid-sensitive drug release and targeted delivery of metallic chemotherapeutic agents [127]. In conclusion, nano-based drug delivery systems can overcome the drawbacks of natural polyphenolic compounds and broaden their application potential in clinical cancer treatment. According to the structural properties of natural polyphenolic compounds, they can be smartly integrated into nano-based drug delivery systems and achieve better antitumor performances in vivo.

## 4. Progress in Nanocarrier-Mediated Delivery of Polyphenolic Compounds in Cancer Therapy

**Figure 5:**
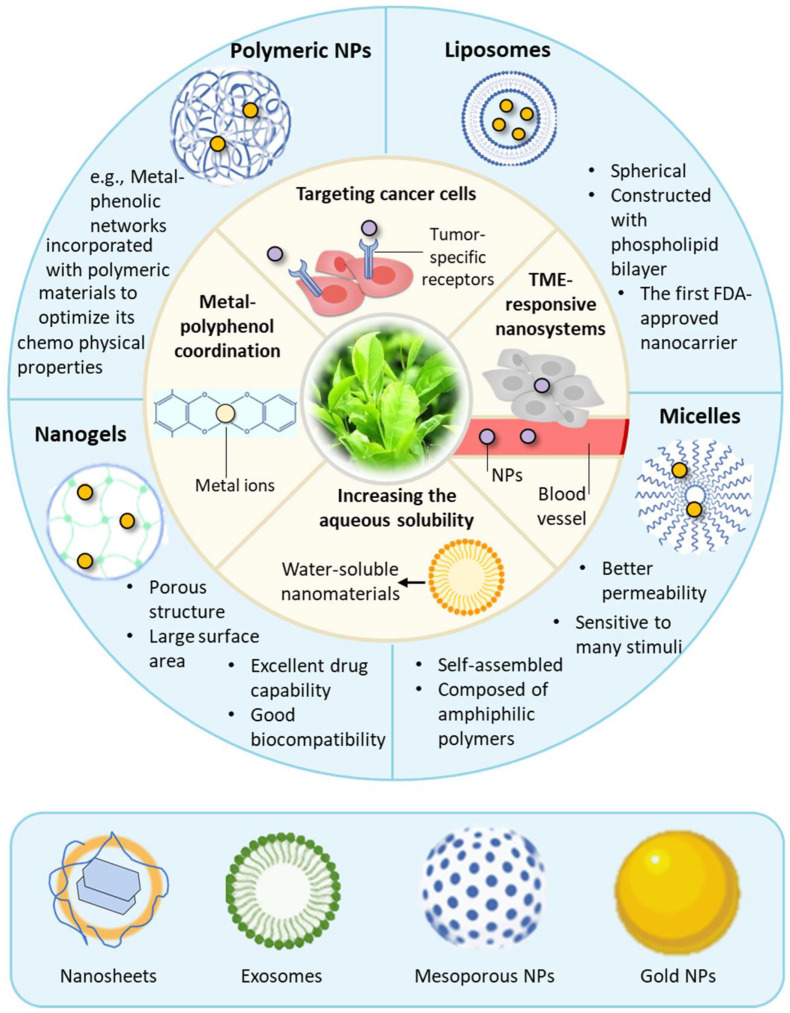
Nano-based drug delivery of natural polyphenolic compounds. Overview of the natural barriers of polyphenolic compounds that nano-based drug delivery systems can overcome (inner ring), and nanocarriers that are generally used for the delivery of natural polyphenolic compounds (outer ring). Nano-based drug delivery systems can achieve targeted delivery of drugs to tumor cells and can specifically respond to chemo-physical stimuli in the TME. Additionally, they can increase the aqueous solubility of polyphenolic compounds. Moreover, we can smartly integrate polyphenolic compounds into nano-based drug delivery systems by taking advantage of their structural properties. Liposomes, micelles, nanogels, and polymeric NPs are four commonly used nanocarriers in the delivery of natural polyphenolic compounds. Bottom box: some other nano-based drug delivery systems of natural polyphenolic compounds in the treatment of cancer.

There have been an increasing number of studies demonstrating promising outcomes in the nanocarrier-mediated delivery of polyphenolic compounds in cancer therapy. In this section, we review the commonly used nanocarriers and their characteristics in the delivery of natural polyphenolic compounds to enhance the therapeutic efficacy of cancer (Table 2, Figure 5).

### 4.1. Liposome-Mediated Delivery of Polyphenolic Compounds

Liposomes, a kind of spherical nanocarrier with a phospholipid bilayer similar to cell membranes, are the first FDA-approved nanocarriers that can be used in clinical treatments [142]. Liposomes are widely used in the development of nanomedicine because of their good biosafety and bioavailability. A tea polyphenol liposome was designed to treat *helicobacter pylori* infection, which is one of the main causes of gastric cancer [143]. The phospholipid layer in this nanoliposome can inhibit the growth of *helicobacter pylori* via fusion with the bacterial membrane [144]. Moreover, tea polyphenols in this nanosystem can reduce inflammation and improve the gut microbes to construct a healthier gastrointestinal environment [144]. This tea polyphenol-based nanoliposome provides a new strategy for treating *helicobacter pylori* infection and preventing the subsequent development of gastric cancer [144]. Some researchers used liposome nanocarriers to achieve synergistic treatment outcomes for polyphenolic compounds and traditional chemotherapeutic drugs. Specifically, the polyphenolic compound resveratrol and chemotherapeutic agent docetaxel were encapsulated in PEGylated liposomes, which can realize the controlled release of drugs and display higher cellular uptake rates in cancer cells [129]. The authors first evaluated the release profile and cytotoxicity of this nanoplatform in prostate cancer PC3 cells [129]. Additionally, in vivo assays demonstrated that the co-delivery of resveratrol and docetaxel can suppress tumor growth by inhibiting cell proliferation and triggering apoptosis, which acted synergistically in treating prostate cancer [129]. Incorporating polyphenolic compounds into liposomes can also enhance their stability. Some researchers discovered that embedding the polyphenolic compounds curcumin or/and EGCG into liposomes can increase their stability in blood circulation [145]. Moreover, these nanoformulations also possessed good anti-cancer performances in prostate cancer cells and bladder cancer cells [145]. Liposomes can also be applied for drug delivery in special physiological environments, such as the gastrointestinal environment. The encapsulation of therapeutic agents by liposomes can improve their stability in these environments and enhance their bioavailability [130]. A kind of eudragit-coated liposome was designed to deliver two naturally occurring compounds resveratrol and artemisinin, which made them more stable in the gastrointestinal environment [130]. Combinational administration of resveratrol and artemisinin showed cytotoxic effects on intestinal adenocarcinoma cells by promoting the generation of ROS, which provided a potential strategy for treating intestinal tumors [130].

### 4.2. Micelles as Nanocarriers for Drug Delivery

Micelles are self-assembled nanocarriers that typically have a hydrophilic polymeric shell and a hydrophobic core [146]. Compared with other nanocarriers, nanomicelles tend to be smaller in size and have better permeability at the lesion sites [147]. Moreover, micelles are sensitive to many endogenous and exogenous stimuli such as pH, hypoxia, light, and temperature, which makes them an ideal nanocarrier for various therapeutic agents [148]. Therefore, they are potential nanocarriers for the targeted delivery and controlled release of polyphenolic compounds. Hypoxia at tumor sites often limits the treatment efficacy of photodynamic therapy [149]. Resveratrol can alleviate this phenomenon by reducing oxygen consumption by tumor cells [131]. Based on this, a tumor-targeted nanomicelle loaded with the hypoxia modulator resveratrol and photodynamic reagent Ce6 was constructed to treat oral squamous cell carcinoma by triggering autophagic cell death and the apoptosis of cancer cells [131]. Moreover, some researchers designed a GSH-sensitive nanomicelle integrated with the polyphenolic compound curcumin to treat esophageal cancer [132]. The release of curcumin was stimulated by GSH in the TME, which improved the delivery efficacy of curcumin to tumor sites [132]. In vivo pharmacokinetic research showed that loading curcumin into nanomicelles can improve the plasma concentration of curcumin and enhance its bioavailability [132]. Application of nanomicelles can also improve the poor solubility of natural polyphenolic compounds. The nano poly (Lactide-co-Glycolide) (PLGA)–curcumin micelle was synthesized to reverse gemcitabine resistance, as curcumin has the ability to suppress the activation of the NF-κB signaling pathway during chemotherapy [133]. Encapsulation into nanomicelles increased the solubility of curcumin up to 10,000-fold, which greatly enhanced their antitumor performance in vivo [133]. Some nanomaterials of micelles not only have low toxicities to healthy tissues but also exhibit therapeutic effects on tumor lesions. Due to their special structural properties, natural polyphenolic compounds have been applied in the synthesis of some therapeutic carrier materials. Based on this, an EGCG-based micelle was constructed, and it could stably deliver drugs to tumor sites [136]. Encapsulated drug synergies with functional EGCG-based micelles can achieve better antitumor outcomes compared with conventional carriers [136].

### 4.3. Drug Delivery Mediated by Nanogels

Nanogels are a kind of nanocarrier with porous structures and large surface/volume ratios that have an excellent ability for encapsulating either hydrophilic or hydrophobic therapeutic agents [150]. Recently, many researchers have attempted to apply nanogels to tumor therapy, as nanogels can effectively improve the permeability and retention times of drugs at tumor sites [151]. A TME-responsive nanogel loaded with resiquimod and the polyphenolic compound EGCG was designed to relieve the immunosuppression in the TME. This nanogel could increase the ratio of cytotoxic T cells to regulatory T cells (Tregs) in tumor sites and inhibit the expression of PD-L1, which significantly improved the efficacy of immunotherapy [137]. Other researchers synthesized a pH- and thermo-responsive nanogel loaded with doxorubicin and curcumin to improve the treatment outcomes of colon cancer [135]. Mechanistically, curcumin can enhance the doxorubicin sensitivity of tumor cells by decreasing the expression of p-glycoprotein, which worked synergistically to achieve better treatment outcomes [135]. Nanogels can also achieve long-term drug release and reduce the distribution of drugs in healthy tissues, improving the therapeutic effects and avoiding the side effects of the drugs. A curcumin-loaded nanogel was synthesized via microemulsion photopolymerization, which was demonstrated to display stronger suppressive effects in tumor growth than free curcumin [134].

### 4.4. Other Nano-Based Drug Delivery Systems

Some researchers designed Fe-doped layered double hydroxide (LDH) nanosheets to encapsulate the polyphenolic compound EGCG [138]. This nanoplatform can precisely release Fe and EGCG in the TME while causing no harm to healthy organs [138]. Ferroptosis induced by Fe and EGCG-triggered apoptosis synergistically inhibited tumor growth in melanoma animal models [138]. Nano-based delivery strategies make possible the combinational use of multiple functional drugs simultaneously. A tumor-targeted nano-framework was fabricated using a platinum-based drug that can induce immunogenic cell death (ICD), and EGCG, a polyphenolic compound that can inhibit the activation of PD-L1 [139]. This nanoplatform can inhibit tumor growth not only by triggering ICD but also by enhancing the infiltration of cytotoxic T cells at the tumor sites [139]. Recently, pure drug nano-assemblies without carriers have gained much attention because they have a very high or even approximately 100% drug loading efficiency, and they are very biocompatible, without carrier-related toxicity [152]. A novel carrier-free nanosystem was constructed based on the self-assembly of the natural products ursolic acid and EGCG [140]. This nanosystem demonstrated satisfactory immunotherapy outcomes in the treatment of hepatocellular carcinoma without any adverse effects on normal tissues [140]. Some plant-derived exosomes rich in polyphenolic compounds also have the potential to be used as nanocarriers for tumor therapy. Exosome-like natural nanovesicles from tea flowers (TFENs) contain various bioactive polyphenolic compounds such as EGCG and epicatechin gallate (ECG) [141]. TFENs can inhibit the proliferation and invasion of breast cancer cells by promoting ROS generation [141]. Moreover, in vivo experiments demonstrated that TFENs have a good targeting ability due to their accumulation in tumor sites and metastatic sites [141]. Intravenous injection or oral administration of TFENs can suppress the progression and metastasis of breast cancer via modulating the gut microbiota [141]. Many nanomaterials can exert tumor suppression functions themselves, such as many metal–organic frameworks (MOFs), D-alpha-tocopheryl poly (ethylene glycol 1000) succinate (TPGS), Pluronic P85, etc. [153,154,155]. Therefore, natural products can synergize with nanomaterials to enhance tumor-suppressive effects. Some researchers developed a synergistic resveratrol nanosystem by using lecithin, a natural phospholipid sourced from soybean and possessing antitumor activity, to achieve excellent tumor-suppressive effects in the treatment of breast cancer [128]. Summarily, nano-based drug delivery systems enhance the bioavailability of natural polyphenolic compounds with poor aqueous solubility, enable the combinational administration of two, or even more, therapeutic agents, and achieve targeted drug delivery to tumor sites with no obvious adverse effects on normal tissues and organs. Therefore, nanocarrier-mediated systems constitute a pivotal arm in the delivery of natural polyphenolic compounds in cancer treatment.

## 5. Conclusions and Perspective

As mentioned above, polyphenolic compounds, one of the most common metabolites found in herb plants, vegetables, and fruits, have been found to display potent antitumor properties via regulating different signaling pathways in various cancer types, presenting potential candidates for the development of antitumor agents. To improve the inherent limitations of pure polyphenolic compounds, such as low stability, weak targeting ability, and poor solubility and bioavailability, nano-based drug delivery systems (e.g., liposome-based, micelle-based, and nanogel-based) have been widely used to achieve targeted delivery and maximize the treatment efficacy of polyphenols. In addition to the encapsulation of polyphenols alone, recent studies also focus on the combinational package of polyphenols and drugs applied to chemotherapy, immunotherapy, and radiotherapy. Due to the synergistic effects displayed by polyphenols and antitumor drugs, these nanoformulations can achieve better treatment outcomes both in vitro and in vivo. As a result, the application of nano-based drug delivery systems can significantly broaden the use of polyphenolic compounds for clinical cancer treatment.

Despite the promising prospects, several concerns should also be addressed to drive the application of nano-based drug delivery systems of polyphenols for treating cancers. First, more nanocarriers should be developed to provide more candidates for packaging polyphenolic compounds as much as possible, which may improve the treatment efficacy of more kinds of polyphenols. In addition, current investigations on the antitumor effects of nano-wrapped drugs are mainly conducted in tumor cell lines and mouse models. More clinical trials should be carried out to verify the treatment effects and safety of nano-packed polyphenols, and there is still a long way to the real use of these nanoformulations in clinical cancer treatment. Furthermore, many nanocarriers have been successfully used to package polyphenols on the laboratory scale, but this may not be realized for scale-up production at the factory level and thus should be taken into consideration in future research.

In summary, nano-wrapped polyphenolic compounds show improved antitumor effects compared with free drugs, which may provide promising candidates for the development of anticancer agents.

## Figures and Tables

**Table 1:** Anti-cancer effects of polyphenolic compounds from different sources and related molecular mechanisms.

Name	Source	Tumor Model	Mechanisms	Ref.
EGCG	Green tea, etc.	Prostate cancer	Targeting the Akt/PI3K pathway to inhibit cell proliferation.	[31]
		Breast cancer	Suppressing the activities of DNMT to promote the expressions of tumor suppressor genes.	[32]
		Myeloma	Downregulating the expressions of miR-25, miR-92, miR-141, and miR-200a to activate the expression of tumor suppressor gene p53.	[33]
		Colorectal cancer	Modulating gut microbial composition.	[34]
PB2	Grape seeds, peanut skin, etc.	Liver cancer; hepatocellular cancer	Targeting the Akt/PI3K pathway to inhibit cell proliferation.	[35]
		Hepatocellular cancer	Targeting the PKM2/HIF-1α signaling pathway to trigger apoptosis and inhibit cell proliferation.	[36]
Resveratrol	Grapes, berries, soybeans, etc.	Breast cancer	Targeting the Wnt/β-catenin signaling pathway to inhibit cell proliferation.	[37]
		Colorectal cancer	Targeting the TNF-β/NF-κB signaling pathway to inhibit cell proliferation.	[38]
		Breast cancer	Suppressing the activity of DNMT to enhance the expression of ATP2A3.	[39]
		Breast cancer; pancreatic cancer; prostate cancer	Inducing apoptosis of cancer cells by suppressing the phosphorylation of the Src-STAT3 signaling pathway.	[40]
		Non-small-cell lung cancer	Modulating the AMPK/mTOR signaling pathway to trigger autophagy.	[41]
		Colorectal cancer	Preventing EMT by inhibiting the TGF-β/Smad signaling pathway.	[42]
Curcumin	Curcuma longa (turmeric)	Cervical cancer	Targeting the NF-κB signaling pathway to inhibit cell proliferation.	[43]
		Lung cancer	Enhancing ROS generation and FOXO3a expression, thereby triggering apoptosis.	[44]
		Prostate cancer	Inducing the apoptosis of CAFs to prevent the growth and metastasis of tumors.	[45]
Genistein	Legumes and dentate plants	Esophageal carcinoma	Targeting the JAK/STAT3 signaling pathway to inhibit cell proliferation.	[46]
		Breast cancer	Decreasing the CpG methylation in the promoters of BRAC1.	[47]
Hispolon	Traditional medicinal mushroom *phellinus linteus*	Breast cancer	Inhibiting the NF-κB signaling pathway and suppressing the expression of MMP-9 to prevent cell invasion.	[48]
Quercetin	Green tea, onion, etc.	Breast cancer	Increasing the acetylation of histone H3K9 in the promoter of BRCA1 (combination effects with curcumin).	[49]
		Gastric cancer	Decreasing the expression of Bcl-2 and triggering apoptosis.	[50]
PE5	Roots of *Phragmipedium* species	Lung cancer	Targeting Akt/mTOR and Bcl-2 signaling pathways to trigger autophagy and apoptosis.	[51]
Luteolin	Celery; chrysanthemum flowers	Breast cancer	Increasing the expressions of p53 and BAX while decreasing the level of Bcl-2, thereby triggering apoptosis.	[52]
Agrimoniin	*Agrimonia pilosa ledeb*	Pancreatic cancer	Increasing intracellular ROS levels and triggering apoptosis.	[53]
HPE	*Hibiscus sabdariffa*	Colon carcinoma	Inhibiting CD44/c-MET signaling pathway to decrease the expression of MMPs, thereby preventing tumor metastasis.	[54]
Gossypol	Cottonseed, etc.	Cervical cancer	Inhibiting the FAK signaling pathway and decreasing the expression of MMPs, thereby preventing tumor metastasis.	[55]
		Cervical cancer	Reversing the EMT mediated by TGF-β.	[55]
Castalagin	Camu-camu (*Myrciaria dubia*)	Non-small-cell lung cancer	Improving the infiltration of CD8+ T cells and enhancing the efficacy of anti-PD-1 therapy by modulating gut microbiota.	[12]

Abbreviations: EGCG, epigallocatechin-3-gallate; Akt, protein kinase B; PI3K, phosphatidylinositide 3-kinase; DNMT, DNA methyltransferase; HDAC, histone deacetylase; miR, microRNA; PB2, proanthocyanidin-B2; PKM2, pyruvate kinase M2; HIF-1α, hypoxia-inducible factor 1α; TNF-β, tumor necrosis factor-β; NF-κB, nuclear factor kappa-B; ATP2A3, sarcoplasmic/endoplasmic reticulum calcium ATPase 3; STAT3, signal transducer and activator of transcription-3; AMPK, adenosine 5′-monophosphate (AMP)-activated protein kinase; mTOR, mammalian target of rapamycin; EMT, epithelial–mesenchymal transition; TGF-β, transforming growth factor-β; ROS, reactive oxygen species; FOXO3a, forkhead box protein O3a; CAFs, cancer-associated fibroblasts; JAK, janus-activated kinase; BRAC1, breast cancer 1; MMPs, matrix metalloproteinases; Bcl-2, B-cell lymphoma-2; PE5, 2-(4″-hydroxybenzyl)-5-2″-dihydroxy-3-methoxystilbene; BAX, Bcl-2-associated X protein; HPE, *Hibiscus sabdariffa* extract; CD44, cluster of differentiation-44; c-MET, cellular-mesenchymal epithelial transition; FAK, focal adhesion kinase; PD-1, programmed death-1.

**Table 2:** Recent progress in nano-based drug delivery systems of natural polyphenolic compounds for cancer therapy.

Nanocarriers/Nanosystem	Natural Products (Therapeutic Agents)	Tumor Model	Therapy Strategies	Ref.
Cyclodextrin-based nanoformulation	Quercetin (ginsenoside Rg3)	Colorectal cancer	Chemotherapy; immunotherapy	[96]
Quercetin–ferrum NPs	Quercetin	Melanoma	Photothermal therapy; immunotherapy	[125]
CaCO_3_ NPs	Kaempferol	Lung cancer	Chemotherapy	[99]
Gold NPs	Genistein	Prostate cancer	Chemotherapy	[102]
Mesoporous silica NPs	Resveratrol	Gastric cancer	Chemotherapy	[103]
Lecithin	Resveratrol	Breast cancer	Chemotherapy	[128]
Liposomes	Resveratrol (docetaxel)	Prostate cancer	Chemotherapy	[129]
Eudragit-coated liposomes	Resveratrol (artemisinin)	Intestinal tumors	Chemotherapy	[130]
Micelles conjugated on hyaluronic nanogel	Resveratrol (Ce6)	Oral squamous cell carcinoma	Chemotherapy; photodynamic therapy	[131]
Micelles	Curcumin	Esophageal cancer	Chemotherapy	[132]
Micelles	Curcumin	Breast cancer	Chemotherapy	[133]
Nanogels	Curcumin	Liver cancer	Chemotherapy	[134]
Nanogels	Curcumin	Colon cancer	Chemotherapy	[110]
Nanogels	Curcumin (doxorubicin)	Colon cancer	Chemotherapy	[135]
Metal–phenolic network	Gossypol (Ce6)	Breast cancer	Chemotherapy; immunotherapy; photodynamic therapy	[121]
Metal–phenolic network	Tannic acid (oxaliplatin)	Colon cancer	Chemotherapy; immunotherapy	[123]
Nanogels	Tannic acid (cisplatin)	/	/	[127]
Nanoassembly	EGCG (siPD-L1)	Liver cancer	Immunotherapy	[119]
Micellar nanocomplex	EGCG (sunitinib)	Kidney cancer	Chemotherapy	[136]
Nanogels	EGCG (resiquimod)	Melanoma	Immunotherapy	[137]
Iron-doped LDH Nanosheets	EGCG	Melanoma	Chemotherapy; chemodynamic therapy	[138]
Platinum NPs	EGCG	Breast cancer	Immunotherapy	[139]
Nanoassembly	EGCG (ursolic acid)	Hepatocellular carcinoma	Immunotherapy	[140]
Exosome-like natural nanovesicles from tea flowers	EGCG, ECG, etc.	Breast cancer	Chemotherapy	[141]

## Data Availability

Not applicable.

## Abbreviations

Akt: protein kinase B; AMPK: adenosine 5′-monophosphate-activated protein kinase; ATP2A3: sarcoplasmic/endoplasmic reticulum calcium ATPase 3; BAX: Bcl-2-associated X protein; Bcl-2: B-cell lymphoma-2; BRAC1: breast cancer 1; CAFs: cancer-associated fibroblasts; CD44: cluster of differentiation-44; CDC2: cell division cycle 2; CDC25C: M-phase inducer phosphatase 3; Ce6: chlorin e6; c-MET: cellular-mesenchymal epithelial transition; DNMT: DNA methyltransferase; ECG: epicatechin gallate; ECM: extracellular matrix; EGCG: epigallocate-chin-3-gallate; EGFR: epidermal growth factor receptor; EIF2α: eukaryotic initiation factors 2α; EMT: epithelial–mesenchymal transition; EPR: enhanced permeability and retention; FAK: focal adhesion kinase; FOXO3a: forkhead box O3; HDAC: histone deacetylase; HIF-1α: hypoxia-inducible factor 1α; HPE: *hibiscus sabdariffa* extract; ICD: immunogenic cell death; JAK1/2: Janus-activated kinase 1/2; LDH: layered double hydroxide; MAPK: mitogen-activated protein kinase; MMPs: matrix metalloproteinases; MOFs: metal–organic frameworks; mTOR: mammalian target of rapamycin; NF-κB: nuclear factor kappa-B; NPs: nanoparticles; PB2: proanthocyanidin-B2; PD-1: programmed death-1; PD-L1: programmed death-ligand 1; PE5: 2-(4″-hydroxybenzyl)-5-2″-dihydroxy-3-methoxystilbene; PEG: polyethylene glycol; PI3K: phosphatidylinositide 3-kinase; PKM2: pyruvate kinase M2; PLGA: poly (Lactide-co-Glycolide); ROS: reactive oxygen species; RTK: receptor tyrosine kinase; STAT3: signal transducer and activator of transcription 3; STING: stimulator of interferon genes; TFENs: exosome-like natural nanovesicles from tea flowers; TfR1: transferrin receptor 1; TGF: transforming growth factor; TME: tumor microenvironment; TNF: tumor necrosis factor; TPGS: D-alpha-tocopheryl poly (ethylene glycol 1000) succinate; Tregs: regulatory T cells.
